# Genomic characterization of two *Faecalibacterium* strains isolated from a healthy Japanese individual

**DOI:** 10.1128/mra.00548-25

**Published:** 2025-07-31

**Authors:** Hidehiro Toh, Yuta Fujimura, Soma Ryuto, Tien Thi Thuy Nguyen, Yusuke Fujii, Hidetoshi Morita, Kensuke Arakawa

**Affiliations:** 1 Advanced Genomics Center, National Institute of Genetics 26359, Mishima, Shizuoka, Japan; 2 Graduate School of Environmental, Life, Natural Science and Technology, Okayama University 12997, Okayama, Japan; 3 College of Agriculture and Forestry, Hue University, Hue, Vietnam; Wellesley College, Wellesley, Massachusetts, USA

**Keywords:** *Faecalibacterium*, human gut microbiota, butyrate-producing bacteria

## Abstract

The genome sequences of two *Faecalibacterium* strains isolated from a healthy Japanese individual were analyzed. Comparative genomics revealed the smallest genome within the genus to date and identified core gene clusters, providing new insights into the minimal genomic requirements and evolutionary adaptation of *Faecalibacterium* species.

## ANNOUNCEMENT

*Faecalibacterium* species are among the most abundant butyrate-producing bacteria in the human gut and play a key role in maintaining gut barrier integrity and modulating inflammation (1). Increasing evidence supports their health-promoting properties and highlights them as promising next-generation probiotics (2). However, genomic information on *Faecalibacterium* species other than *Faecalibacterium prausnitzii* remains limited, especially in Asian populations (3, 4). To address this gap and to better understand the diversity and functional potential of *Faecalibacterium* in the Japanese gut, we isolated two novel strains, F15 and a30, from a healthy individual and determined their draft genome sequences.

A fecal sample from a healthy 22-year-old Japanese man from Okayama, Japan, was serially diluted under anaerobic conditions and plated on modified GAM agar containing 1% pectin. After 48 hours of anaerobic incubation at 37°C, colonies with typical *Faecalibacterium* morphology were selected and identified via 16S rRNA sequencing using primers 27F (5′-AGAGTTTGATCMTGGCTCAG-3′) and 1525R (5′-AAGGAGGTGATCCAGCC-3′) (Eurofins) on a 3730xl DNA Analyzer (Applied Biosystems) (5). The isolates were cultured in modified GAM broth with 0.5% sodium acetate at 37°C for 24 hours anaerobically. Cells were harvested, washed, and treated sequentially with lysozyme, achromopeptidase, proteinase K, and SDS. Genomic DNA was extracted using phenol:chloroform:isoamyl alcohol, purified by ethanol precipitation and centrifugation, treated with RNase, further purified using NaCl and PEG precipitation, washed with ethanol, and resuspended in TE buffer (6).

**Fig 1:**
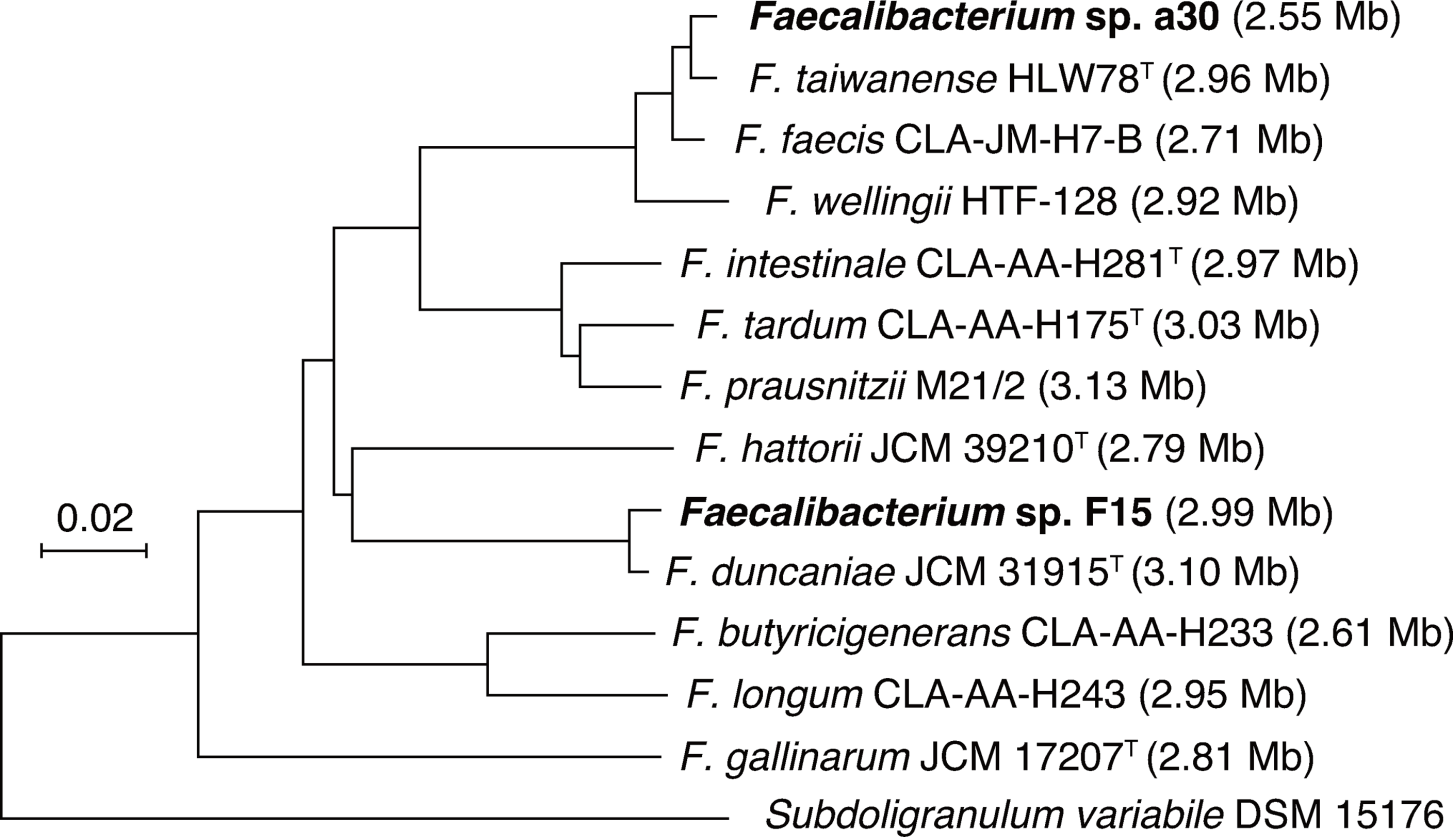
Neighbor-joining phylogenetic tree based on the *recA* gene sequences showing the relationships between the two analyzed strains and related *Faecalibacterium* species. The *recA* gene sequences for each species were obtained from reference genomes available at NCBI. Multiple sequence alignment was performed using ClustalW2, and the phylogenetic tree was constructed using MEGA11 (11). Values in parentheses indicate genome size in megabases. The scale bar indicates the number of nucleotide substitutions per site.

Genomic libraries were prepared using the Nextera XT DNA Library Prep Kit (Illumina) and sequenced with 300-base paired-end reads on the MiSeq platform using the MiSeq Reagent Kit v3 (Illumina). Sequencing reads were quality filtered to remove low-quality bases and short reads, and subsequently assembled *de novo* using CLC Genomics Workbench v10.1.1 with default parameters. Protein-coding genes were annotated using DFAST v1.6.0 (7). A summary of the sequencing and genome assembly statistics is presented in Table 1. For species-level classification of *Faecalibacterium*, the *recA* gene proved more reliable than the 16S rRNA gene as a phylogenetic marker (8). The *recA* sequence of strain F15 showed 99.1% identity to *Faecalibacterium duncaniae* JCM 31915^T^ (9) and an average nucleotide identity (ANI) of 96.7%, supporting its classification as *F. duncaniae* (Fig. 1). Strain a30 showed 99.1% *recA* sequence identity to *Faecalibacterium taiwanense* HLW78^T^ (10) and an ANI of 97.8%, consistent with classification as *F. taiwanense* (Fig. 1).

**TABLE 1:** Genomic features of two *Faecalibacterium* strains analyzed in this study

Species	Strain	No. of reads	No. of contigs	Genome size (bp)	*N*_50_ contig size (bp)	GC content (%)	Genome coverage (×)	No. of protein-coding genes
*F. duncaniae*	F15	550,916	141	2,988,843	44,918	56.2	55.3	2,791
*F. taiwanense*	a30	481,940	89	2,554,504	55,053	56.6	56.6	2,308

Remarkably, strain a30 has the smallest genome (2.55 megabases) among all *Faecalibacterium* species reported to date. To estimate the core genome of the genus, we compared the gene clusters of strain a30 with those of 11 other reference strains representing *Faecalibacterium*. A total of 1,203 genes were shared by all 12 genomes and were inferred to constitute the core gene cluster of *Faecalibacterium* species. These findings provide new insights into the minimal genomic requirements for the genus and establish a foundation for understanding its essential functions and evolutionary adaptations.

## Data Availability

This study is associated with the BioProject registered under accession number PRJDB15359. Draft genome sequences for the two strains (F15 and a30) have been submitted to the DDBJ/GenBank/EMBL databases under the accession numbers BSSS01000001–BSSS01000141 and BSSR01000001–BSSR01000089, respectively. The raw sequencing data are available in the Sequence Read Archive under accession number DRA020529.

## References

[1] Singh V, Lee G, Son H, Koh H, Kim ES, Unno T, Shin JH. 2022. Butyrate producers, “The Sentinel of Gut”: their intestinal significance with and beyond butyrate, and prospective use as microbial therapeutics. Front Microbiol 13:1103836. doi:10.3389/fmicb.2022.1103836 36713166 PMC9877435

[2] Martín R, Rios-Covian D, Huillet E, Auger S, Khazaal S, Bermúdez-Humarán LG, Sokol H, Chatel JM, Langella P. 2023. Faecalibacterium: a bacterial genus with promising human health applications. FEMS Microbiol Rev 47:fuad039. doi:10.1093/femsre/fuad039 37451743 PMC10410495

[3] Lindstad LJ, Lo G, Leivers S, Lu Z, Michalak L, Pereira GV, Røhr ÅK, Martens EC, McKee LS, Louis P, Duncan SH, Westereng B, Pope PB, La Rosa SL. 2021. Human gut Faecalibacterium prausnitzii deploys a highly efficient conserved system to cross-feed on β-mannan-derived oligosaccharides. MBio 12:e0362820. doi:10.1128/mBio.03628-20 34061597 PMC8262883

[4] Ueda A, Shinkai S, Shiroma H, Taniguchi Y, Tsuchida S, Kariya T, Kawahara T, Kobayashi Y, Kohda N, Ushida K, Kitamura A, Yamada T. 2021. Identification of Faecalibacterium prausnitzii strains for gut microbiome-based intervention in Alzheimer’s-type dementia. Cell Rep Med 2:100398. doi:10.1016/j.xcrm.2021.100398 34622235 PMC8484692

[5] Okubo T, Ikeda S, Yamashita A, Terasawa K, Minamisawa K. 2012. Pyrosequence read length of 16S rRNA gene affects phylogenetic assignment of plant-associated bacteria. Microbes Environ 27:204–208. doi:10.1264/jsme2.me11258 22791055 PMC4036018

[6] Wilson K. 2001. Preparation of genomic DNA from bacteria. Curr Protoc Mol Biol Chapter 2:Unit doi:10.1002/0471142727.mb0204s56 18265184

[7] Tanizawa Y, Fujisawa T, Nakamura Y. 2018. DFAST: a flexible prokaryotic genome annotation pipeline for faster genome publication. Bioinformatics 34:1037–1039. doi:10.1093/bioinformatics/btx713 29106469 PMC5860143

[8] Tanno H, Chatel JM, Martin R, Mariat D, Sakamoto M, Yamazaki M, Salminen S, Gueimonde M, Endo A. 2023. New gene markers for classification and quantification of Faecalibacterium spp. in the human gut. FEMS Microbiol Ecol 99:fiad035. doi:10.1093/femsec/fiad035 36990641 PMC10093996

[9] Sakamoto M, Sakurai N, Tanno H, Iino T, Ohkuma M, Endo A. 2022. Genome-based, phenotypic and chemotaxonomic classification of Faecalibacterium strains: proposal of three novel species Faecalibacterium duncaniae sp. nov., Faecalibacterium hattorii sp. nov. and Faecalibacterium gallinarum sp. nov. Int J Syst Evol Microbiol 72. doi:10.1099/ijsem.0.005379 35416766

[10] Liou JS, Zhang WL, Hsu LW, Chen CC, Wang YT, Mori K, Hidaka K, Hamada M, Huang L, Watanabe K, Huang CH. 2024. Faecalibacterium taiwanense sp. nov., isolated from human faeces. Int J Syst Evol Microbiol 74:006413. doi:10.1099/ijsem.0.006413 38848117 PMC11261667

[11] Tamura K, Stecher G, Kumar S. 2021. MEGA11: molecular evolutionary genetics analysis version 11. Mol Biol Evol 38:3022–3027. doi:10.1093/molbev/msab120 33892491 PMC8233496

